# A comparison between the self-report of chronic cardiovascular diseases with health insurance data: insights from the population-based LIFE-Adult study

**DOI:** 10.1186/s13690-025-01606-3

**Published:** 2025-05-07

**Authors:** Samira Zeynalova, Peter Worringen, Stefan Bassler, Anja Martin, Katrin Czech, Lars Greulich, Matthias Reusche, Ute Enders, Nigar Reyes, Maryam Yahiaoui-Doktor, Matthias Collier, Markus Loeffler, Tina Stegmann

**Affiliations:** 1 Institute for Medical Informatics, Statistics and Epidemiology (IMISE), Leipzig University, 04107 Leipzig, Germany; 2 Leipzig Research Centre for Civilization Diseases (LIFE), 04103 Leipzig, Germany; 3 AOK PLUS DE, 01067 Dresden, Germany; 4 IKK classic, 01099 Dresden, Germany; 5 The Clinical Trial Centre (ZKS) Leipzig, Leipzig University, 04107 Leipzig, Germany; 6 Department of Cardiology, Leipzig University Hospital, 04103 Leipzig, Germany

**Keywords:** Population-based, Health insurance, Self-report, Validation, Agreement, Heart failure, Stroke, Atrial fibrillation, Myocardial infarction

## Abstract

**Background:**

Self-reporting is a common approach in observational epidemiological studies. However, information can be biased by several causes and can, therefore, affect the outcomes of the investigations. This analysis aimed to evaluate the agreement between self-reported data from a population-based cohort study with data from two large German health insurance companies.

**Methods:**

Participants with available self-reported diagnoses of a history of stroke, atrial fibrillation (AF), heart failure (HF), and myocardial infarction (MI) from the baseline and the follow-up (after six years) surveys of the prospective population-based LIFE-Adult study were included in this study. Two health insurance companies provided ICD-10-GM codes. The agreement between the self-reports and health insurance data (HID) was examined by calculating sensitivity, specificity, Cohen`s Kappa, positive and negative predictive values. We used multivariable logistic regression models to examine whether odds ratios (OR) for the association between risk factors and the certain disease changed, depending on whether self-reports or HID was used as the dependent variable.

**Results:**

One thousand seven hundred eighty four individuals with complete data were included in this interim analysis. Mean age was 58 (SD±12) years and 984 (55%) were female. 52 (2.9%) subjects reported a history of stroke, 99 (5.6%) AF, 63 (3.5%) HF, and 46 (2.6%) MI. Compared with the HID, a high specificity was found for all four diagnoses (stroke: 99% [95% CI 99.3-99.9]; AF: 99% [95% CI 98.1-99.2], HF: 98% [95% CI 97.6-98.9], and MI: 99% [95% CI 98.9-99.7]). Sensitivity ranged from 58% (95% CI 47.4-69.5) for stroke over 61% (95% CI 48.8-74.0) for MI, to 65% (95% CI 56.6-73.9) for AF. Sensitivity in HF was the lowest (20% [95% CI 14.4-26.5]).

**Conclusion:**

The use of German health insurance data is a feasible method for verifying population-based self-reported diagnoses. The sensitivity varied among the self-reported diseases compared with the health insurance data, whereas the specificity was continuously high. The verification of self-reported diagnoses using health insurance data as an additional data source may be considered in future population-based assessments to reduce misclassification error of self-reported data.

**Supplementary Information:**

The online version contains supplementary material available at 10.1186/s13690-025-01606-3.

**Table:** 

**Text box 1. Contributions to the literature**
(I) In cardiovascular healthcare research, self-reported data from population-based studies are commonly used to estimate the prevalence of diagnoses. However, self-reports may contain incorrect information and can bias the results.
(II) This study evaluated the agreement between self-reported data of the diagnoses stroke, atrial fibrillation, myocardial infarction, and heart failure and data from two German statutory health insurance companies. This new approach did show its feasibility within the German data protection regulations.
(III) A high agreement for stroke, atrial fibrillation, and myocardial infarction was found whereas low agreement for heart failure was observed.
(IV) Multiple data sources may be considered in population-based studies, as relying on a single one may lead to different results. In Germany, health insurance data may be considered as an additional data source for confirming self-reported diagnoses, particularly for the diagnosis of heart failure.

## Introduction

Cardiovascular diseases (CVD) are the leading cause of death globally. Ischemic heart disease, stroke, and atrial fibrillation (AF) are particularly associated with high mortality and morbidity rates [1]. They represent a crucial economic burden on the health care system [1]. In 2008, 36.9 billion euros were spent on CVD in Germany, reflecting 14.5% of the total costs in the German healthcare system [2]. Subsequently, sufficient preventive strategies and effective medical therapies are essential to reduce the overall CVD burden and mortality and morbidity rates.

Epidemiological and medical studies commonly use self-reports to gather data and information on prevalent or incident diagnoses [3, 4]. For this method, no interference from a physician or researcher is required. Given the wide usage of self-reported data in epidemiological studies, the accuracy of self-reported data is crucial to avoid bias or errors, e.g., estimates of associations or over- or underestimation of risk parameters. The self-reported data are considered a valuable and cost-effective method for assessing the prevalence and incidence of CVD and associated risk factors in the absence of specific population registers [5]. However, various studies suggest that self-reports can be biased for several reasons [6–8]. For example, sociodemographic factors, understanding of the disease, perception of the disease, severity of symptoms, social desirability, and individual resources can affect the accuracy of the data [4, 9, 10]. In cardiovascular epidemiology, it has been reported that the agreement between self-reports and medical records generally varies (k-statistic: 0.46 for heart failure to 0.8 for myocardial infarction), and validation of patient self-reported data remains challenging [3, 11]. It is, therefore, of epidemiological, health-related research, and clinical interest to understand and validate the accuracy of participant reports and how to interpret population-based generated data.

In the German healthcare system, decentralised and facility-based data processing systems are commonly used, leading to disparate data. Due to the large number of health insurers, no uniform data analysis is possible [12]. This study aimed to investigate the accuracy of self-reported cardiovascular disease in a population-based cohort. We used the example of the German prospective and population-based LIFE-Adult cohort study (Leipzig Research Center for Civilization Diseases). Health insurance data (HID) from two large German large statutory health insurance served as a reference. The following four common cardiovascular diseases, stroke, AF, heart failure (HF), and myocardial infarction (MI), were selected for this analysis. A secondary objective was to demonstrate the feasibility of using self-reported data alongside HID as a means of comparison in compliance with German security and data protection regulations.

## Material and methods

### Study population

The LIFE-Adult study is a large prospective population-based cohort study. A detailed study protocol has previously been published by Loeffler et al. 2015 [13]. The LIFE-Adult study randomly selected 10,000 residents of Leipzig (Saxony, Germany) between 18 and 79 years of age, and represents an age and gender-stratified cohort. All participants underwent a comprehensive health examination at baseline between 2011 and 2014, including health questionnaires and physical and biochemical laboratory examinations [13]. The baseline examinations took place at the study center in Leipzig. The first follow-up survey was conducted between January 1^st^ 2017 and December 31^st^ 2020. It was a questionnaire-based postal survey of all participants, who were asked to complete a health questionnaire. All participants provided written and signed consent forms at baseline. The study was approved by the local ethics committee (*“Ethik-Kommission an der Medizinischen Fakultät der Universität Leipzig”*) and is in accordance with the Declaration of Helsinki. The LIFE Research Center of the Medical Faculty of the University of Leipzig operates on the basis of a data protection concept that aims to comply with data protection regulations within the existing organisational structure of the institute. The security and protection of personal data, medical information, diagnoses, and social data has been regulated in accordance with the requirements of Section 9 BDSG (“*Bundesdatenschutzgesetz”*; Federal Data Protection Act) and its appendix and Section 78a SGB X (“*Sozialgesetzbuch”;* Social Security Code) and its appendix. For health insurance data, applications were made in accordance with Section 75 SGB X in conjunction with Section 98 SGB. All analyses in connection with secondary data are generally carried out in a pseudonymized form. Publication will be in anonymized form only. We accessed data for research purposes between September 2020 and November 2020. This applies to both the LIFE-Adult study and the health insurance data.

**Fig. 1:**
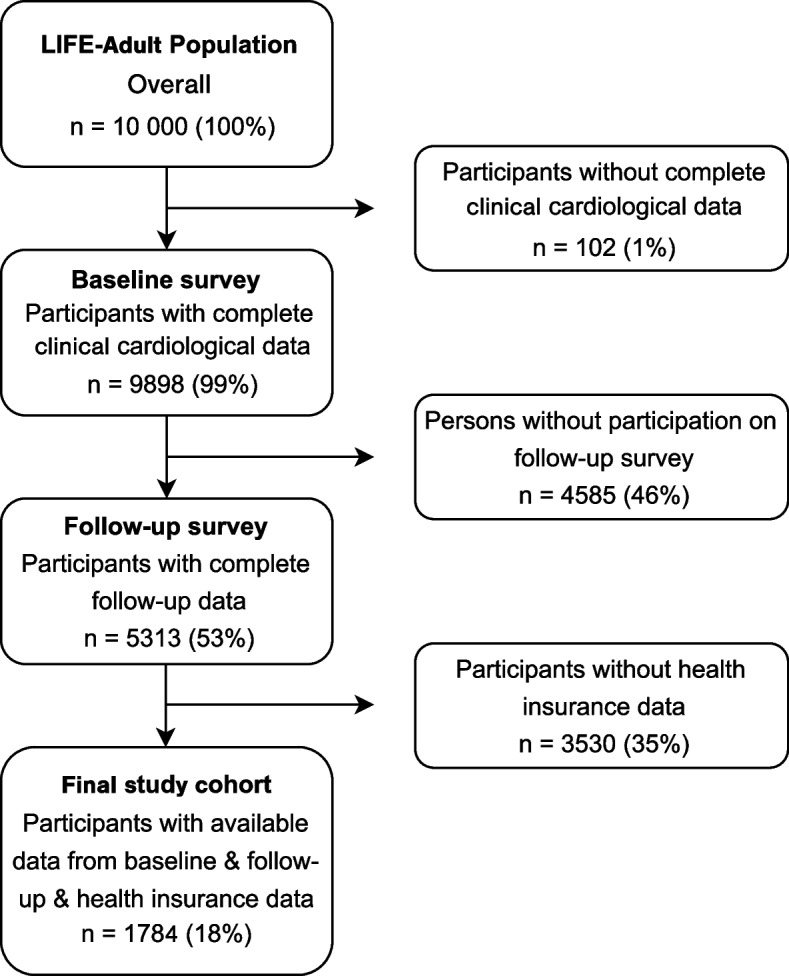
Study flow diagram. Selection of study participants for the final study analysis set

This study presents an interim analysis. We used data from two large statutory health insurance companies. In 9,898 out of the initial 10,000 participants, complete baseline cardiac self-reported information was available. At the time of this interim analysis, corresponding information from the follow-up survey was available in 5,313 subjects. Among those with complete baseline and follow-up data, information from the health insurance companies was additionally available for a total of 1,784 participants, since data were provided from two health insurance companies and not all subjects were insured by the same. Therefore, until June 30^th^, 2020, only a subgroup of the total LIFE-Adult study participants was analyzed, Fig. 1.

### Data collection - LIFE-Adult study

Participants were asked for their medical diagnoses according to a standardized and structured questionnaire covering more than 70 common diseases. The year of initial diagnosis and whether a specific treatment was received in the last twelve months was requested. Using MI as an example, questions were structured as follows: "In which year was a heart attack diagnosed for the first time?", "Are you currently being treated for a heart attack?", "Have you ever had a heart attack diagnosed by a doctor?", "How old were you at the time of the heart attack?". Responses were given by “yes” or “no” and the date of the diagnosis was recorded. In the case of several events, the first one was documented. For this interim analysis, we focused on the following four major CVD, stroke, AF, HF, and MI. AF was not assessed at the baseline investigations, but at follow-up.

The baseline study was conducted between the end of 2011 and 2014. The follow-up phase was from the end of 2017 to 2020. Thus, both periods lasted around 3.5 years. Participants were contacted for follow-up approximately six years after the baseline examination. Therefore, the time interval between the baseline and follow-up examinations was largely equal for all participants.

### Data collection - Health care insurance companies

Germany does not have central registries in which individuals'diagnoses and healthcare information are systematically stored and from which they could be provided [12]. However, professionals in the hospitals encode a reliable diagnosis in accordance with the “International Statistical Classification of Diseases and Related Health Problems 10th Revision” (ICD-10). ICD-10 German modification (ICD-10-GM) is the official classification for the encoding of diagnoses in inpatient and outpatient medical care in Germany [14]. The ICD-10-GM code or a certain diagnosis is recorded by the individual health care insurance company. Every person with statutory health insurance has an individual health insurance number, which remains the same even if they change health insurer and enables precise allocation [15]. The “Allgemeine Ortskrankenkasse” (AOK Plus DE) and the “Innungskrankenkasse” (IKK classic) are two large German statutory health insurance companies. The AOK Plus DE and IKK classic provided the ICD-10-GM diagnoses and information on the four selected diseases of the respective subjects from the outpatient and inpatient sectors. Both the ICD diagnosis codes from the inpatient and outpatient sectors were used and evaluated together. Details about the used ICD-10-GM codes are demonstrated in the supporting information Supplemental Table 1. Due to the German healthcare system, HID was postulated as the most reliable source and were chosen as a corresponding data source to investigate the accuracy of the self-reports. Diagnoses are generally classified into four categories within the HID: suspected, confirmed diagnosis, condition after, and exclusion [16]. Within the present study, only the types "confirmed diagnosis" and "condition after" were considered for analyses. There were no solely suspected diagnoses. The data transfer to the health insurance companies was carried out with the help of record linkage and a data pseudonymization procedure, to protect personal data.

**Table 1:** Characteristics of the overall LIFE-Adult study and the final studied subgroup cohort

Characteristics^*^	**Overall LIFE-Adult study** (*n*= 9898)			**Final study cohort** (*n*= 1784)		
	**Male** (*n*= 4711)	**Female** (*n*= 5187)	**Total**	**Male** (*n*= 800)	**Female** (*n*= 984)	**Total**
Mean age, yrs (SD)	58 (± 13)	57 (± 12)	57 (± 12)	58 (± 12)	57 (± 12)	58 (± 12)
BMI, kg/m^2^ (SD)	27.6 (± 4)	27.1 (± 5)	27.2 (± 5)	27.6 (± 4)	27.1 (± 5)	27.3 (± 5)
Active Smoker (%)	1066 (23)	996 (19)	2062 (21)	171 (21)	155 (16)	326 (18)

*BMI* = Body-mass-index

^*^Values are presented as absolute numbers (%) or as mean (± standard deviation; SD)

Detailed information on data linkage is provided in the Supplemental material – Detailed methods.

### Statistical analysis

The self-reports from the follow-up surveys of the LIFE-Adult study were used for the analysis of the agreement between the LIFE-Adult questionnaire data and HID. Only the year and quarter of the diagnosis in the outpatient sector is recorded in the database of the HID. For inpatient treatments, the exact date is available. However, the date of the diagnosis is not listed separately, but only the respective treatment case. Only diseases known at the time of the follow-up interview were included. If a diagnosis was made later in the HID, it was not considered.

Data from 1,784 subjects were analyzed by crosstabulation. The HID were used as a reference to the self-reports. Thus, the numbers for true positive (TP: ‘yes’ in self-reports and in HID), false positive (FP: ‘yes’ in self-reports, but not in HID), true negative (TN: ‘no’ in self-reports and in HID) and false negative (FN: ‘no’ in self-reports, but yes in HID) values were calculated. Specificity (TN/FP+TN), sensitivity (TP/TP+FP), positive predictive value (PPV: TP/TP+FP), and negative predictive value (NPV: TN/FN+TN) were calculated. PPV and NPV served as statistical quality criteria to compare the self-reports at the time of the follow-up survey with the HID [17]. The PPV and NPV refer exclusively to the analyzed subgroup of the LIFE-Adult study population.

The agreement of self-reported prevalent cases of stroke, AF, HF, and MI was also assessed by calculating Cohen's kappa [18]. This was suggested by Landis and Koch in 1977: A kappa value of 0.40 is considered fair to poor agreement, a value of 0.41 to 0.60 is considered moderate agreement, a value of 0.61 to 0.80 is considered substantial agreement, and a value of 0.81 to 1.00 is considered excellent agreement [19].

We performed multivariable logistic regression models to examine whether odds ratios (OR) for the association between risk factors and the certain disease changed, depending on whether self-reports or HID was used as the dependent variable in the respective model. For the multivariable regression analyses, data from the follow-up questionnaires of the LIFE-Adult study were used.

HID, including ICD diagnoses, were available from 2013 onwards. If cardiovascular conditions such as MI, stroke, HF, or AF occurred in 2011 or 2012 (which is relevant for participants with baseline examinations from these years), they were still recorded in the HID from 2013 onwards, as people with these conditions usually sought follow-up care, and the corresponding ICD codes were documented.

This analysis was performed to exemplarily evaluate how risk factor analyses may differ based on the underlying data source. For this exemplary analysis four common risk factors were used age, sex, body-mass-index (BMI) > 25 kg/m^2^, and smoking status as independent variables in multivariable logistic regression analyses. The frequency of the dependent variable was based on whether the data were used from the self-report at the time of the follow-up or the HID. Results are presented as OR with a 95% confidence interval (95% CI). Statistical significance was set at *p* < 0.05 (two-sided). Additionally, to evaluate whether misclassification errors in self-reported data introduce bias in an epidemiological study, the covariates of age and sex were used to provide an example of the potential for the self-reported data to produce bias, given the reported sensitivity and PPV. All analyses were performed using IBM SPSS Statistics Version 28.0.1.1(15) Windows (IBM, Chicago, USA).

## Results

Until June 2020, for 1,784 (18%) out of the 10,000 LIFE-Adult study participants, both data sources follow-up questionnaires and HID were available. Selection of participants for final analysis is shown in Fig. 1. The characteristics of the overall LIFE-Adult cohort with complete cardiological data (n= 9,898) and the analyzed subgroup (*n*= 1,784) are depicted in Table 1. We present the characteristics at the time of the follow-up investigation since these data were used for the comparison with the HID. The mean age of the overall LIFE-Adult study cohort was 57 (standard deviation [SD] ± 12) years and 5187 (52%) were female. The mean body mass index (BMI) was 27.2 (± 5) kg/m^2^ and 2,062 (21%) were active smokers. The mean age of the final analyzed LIFE-Adult subgroup was 58 (± 12) years, 984 (55%) were female. The mean BMI was 27.3 (± 5) kg/m^2^ and 326 (18%) were active smokers at the time of the follow-up survey.

The prevalence of the four analyzed diseases is shown in Table 2, stratified by data source. In the follow-up survey 52 (2.9%) of the LIFE-Adult study participants reported a previous history of stroke, 99 (5.5%) had AF, 63 (3.5%) HF, and 46 (2.6%) MI. Compared to available HID, stroke was prevalent in 108 (6.1%) cases, 138 (7.7%) had AF, 171 (9.6%) HF, and 75 (4.2%) MI.

**Table 2:** Descriptive data for the prevalence of stroke, atrial fibrillation, heart failure, and myocardial infarction

	**Overall LIFE-Adult Study** (*n*= 9,898)	**Final Study cohort** (*n*= 1,784)		
	**Baseline**	**Baseline**	**Follow-up**	**HID**
Stroke (%)	219 (2.2)	33 (1.9)	52 (2.9)	108 (6.1)
Atrial fibrillation (%)	not recorded	not recorded	99 (5.5)	138 (7.7)
Heart failure (%)	183 (1.9)	22 (1.2)	63 (3.5)	171 (9.6)
Myocardial infarction (%)	251 (2.5)	32 (1.8)	46 (2.6)	75 (4.2)

*HID* Health insurance data

### Agreement between self-reported and health insurance data

The results of the conformity analysis between the follow-up survey and HID for stroke, AF, HF, and MI are summarized in Table 3. The specificity was 99% (95% CI 99.3–99.9) among stroke, AF, and MI. Specificity for reported HF was 98% (95% CI 97.6–98.9). Sensitivity ranged from 20% (95% CI 14.4–26.5) for HF to 65% (95% CI 56.6–73.9) for AF. The PPV was lowest for HF with 56% (95% CI 43.3–67.8) and highest for stroke with 87% (95% CI 77.3–95.8). For the NPV, the values ranged from 92% (95% CI 90.8–93.4) for HF to 99% (95% CI 98.2–99.2) for MI. Similar results were found for Cohen's kappa. Relatively good agreement was found for stroke (68%), AF (69%) and MI (67%). Poor agreement was found for HF (26%).

**Table 3:** Analyses of the agreement between the self-reports and the health insurance registered diagnoses. Data are presented for the final studied cohort including sensitivity, specificity, positive predictive, and negative predictive values

	Total number of cases^*^	Number of cases separately^**^				**Sensitivity**^**a**^ (95% CI)	**Specificity**^**a**^ (95% CI)	**PPV**^**a**^ (95% CI)	**NPV**^**a**^ (95% CI)	**C. Kappa**^**a**^
		**TP**	**FN**	**FP**	**TN**					
Stroke	1696	45 (2.6)	33 (1.9)	7 (0.4)	1611 (93.7)	58 (47.4–69.5)	99 (99.3–99.9)	87 (77.3–95.8)	98 (97.3–98.7)	68
AF	1784	77 (4.3)	41 (2.3)	22 (1.2)	1644 (92.2)	65 (56.6–73.9)	99 (98.1–99.2)	78 (69.6–86)	98 (96.8–98.3)	69
HF	1784	35 (2.0)	136 (7.6)	28 (1.6)	1585 (88.8)	20 (14.4–26.5)	98 (97.6–98.9)	56 (43.3–67.8)	92 (90.8–93.4)	26
MI	1729	35 (2.0)	22 (1.3)	11 (0.6)	1661 (95.1)	61 (48.8–74)	99 (98.9–99.7)	76 (63.8–88.4)	99 (98.2–99.2)	67

*RP* True positive, *FN* False negative, *FP* False positive, *TN* True negative, *PPV* Positive predictive value, *NPV* Negative predictive value, *95% CI* = Confidence interval (%), *AF* Atrial fibrillation, *HF* Heart failure, *MI* Myocardial infarction

^*^Total number of cases varies due to partly missing values

^**^Values are presented as absolute values (%)

^a^Numbers are presented in %

### Multivariable logistic regression analysis

The exemplary comparison of the associations of predictors for stroke, AF, HF, and MI between the self-reported data versus HID is presented in Table 4. The results for the pre-selected risk factors differed, depending on the primary database. Age was the only pre-defined factor with an independent and significantly associated prediction of all four diseases, and the ORs between both data sources were numerically comparable. Active smoking was not independently associated with either of the four selected conditions, regardless of which data source was used. In the HF model, the OR for BMI > 25 kg/m^2^ was almost doubled when using the self-reported data compared with HID (OR 4.03, 95 % CI 1.43–11.36, *p*= 0.008 vs. OR 2.24, 95 % CI 1.32 to 3.78, *p*= 0.003). In the AF model, the OR for BMI was almost equal, independently which data source was used (HID: OR 1.91, 95% CI [1.08 to 3.41], *p*= 0.027 vs. self-reports: OR 1.85, 95% CI [1.00 to 3.40], *p*= 0.05). Female sex numerically reduced the risk for all four selected conditions. However, the OR and the statistical significance varied depending on whether HID or self-reported data were used (Table 4).

**Table 4:** Multivariable logistic regression models for the assessment of the association between pre-defined risk factors with stroke, atrial fibrillation (AF), myocardial infarction (MI), and heart failure (HF), respectively

**Disease**		Self-reported data^*^		Health insurance data^*^	
		**OR (95% CI)**	***p*-value**	**OR (95% CI)**	***p*-value**
**Stroke**	Age	1.05 (1.01–1.08)	0.004	1.06 (1.04–1.08)	< 0.001
	Sex, female	0.76 (0.41–1.40)	0.375	0.55 (0.36–0.85)	0.006
	Active smokers	0.61 (0.26–1.81)	0.450	1.17 (0.65–2.08)	0.603
	BMI > 25 kg/m^2^	1.09 (0.55–2.16)	0.815	1.07 (0.66–1.74)	0.773
**Atrial fibrillation**	Age	1.08 (1.05–1.12)	< 0.001	1.10 (1.07–1.13)	< 0.001
	Sex, female	0.61 (0.39–0.95)	0.027	0.70 (0.47–1.05)	0.083
	Active smokers	0.84 (0.39–1.82)	0.658	0.86 (0.41–1.79)	0.681
	BMI > 25 kg/m^2^	1.85 (1.00–3.40)	0.050	1.91 (1.08–3.41)	0.027
**Heart failure**	Age	1.09 (1.05–1.14)	< 0.001	1.09 (1.06–1.12)	< 0.001
	Sex, female	0.77 (0.45–1.34)	0.359	0.69 (0.48–0.98)	0.039
	Active smokers	1.01 (0.38–2.65)	0.988	1.23 (0.69–2.19)	0.493
	BMI > 25 kg/m^2^	4.03 (1.43–11.36)	0.008	2.24 (1.32–3.78)	0.003
**Myocardial infarction**	Age	1.05 (1.01–1.08)	0.006	1.04 (1.02–1.07)	< 0.001
	Sex, female	0.18 (0.08–0.41)	< 0.001	0.38 (0.22–0.64)	< 0.001
	Active smokers	1.22 (0.54–2.77)	0.640	0.78 (0.37–1.63)	0.508
	BMI > 25 kg/m^2^	1.74 (0.75–4.01)	0.197	1.11 (0.62–1.97)	0.729

^*^Data are presented as odds ratio (OR) with 95 % confidence interval (95% CI)

Results on misclassification errors in self-reported data based on the covariates age and sex are depicted in the Supplemental Table 2. A higher sensitivity for all reported cardiovascular diseases was observed in men compared to women. The sensitivity was also higher in participants less than 60 years compared to ≥ 60 years. The results for the PPV were almost equal (Supplemental Table 2).

## Discussion

The study provides the following main findings. (I) The use of health insurance-recorded ICD-10-GM codes to verify self-reported data from a population based cohort study led to differences in the agreement of the pre-specified analyzed diagnosis. We found a high agreement for self-reporting of stroke, atrial fibrillation, and myocardial infarction compared with HID-recorded ICD-10-GM codes. In contrast, a poor agreement for the diagnosis of heart failure was observed. (II) Self-reports were associated with underreporting compared to HID. (III) According to German data privacy regulations, the use of health insurance data was a feasible method for verifying the accuracy of self-reported diagnoses in a population-based cohort. (IV) These results support the concept that it is crucial to use multiple data sources, as relying on a single one may lead to different results.

### Agreement between self-reported diagnoses and HID provided data

In this study, we used ICD-10 diagnosis codes from HID to assess the incidence of CVD. We acknowledge that these codes may have limitations compared with a gold standard based on medical record review and standardized event assessment protocols. Therefore, we performed cross-validation and used Cohen's kappa value to assess the agreement between self-reported diagnoses and HID. Although the kappa values were relatively high, they should not be considered 'perfect' due to differences in data collection methods and possible reporting bias. Therefore, kappa values should be interpreted with caution in this context as well.

Stroke showed the highest overall agreement with a specificity of 99%. The self-reported diagnosis of stroke had the third-best sensitivity overall (58%) in our study but was lower compared to previous studies. In contrast, the PPV of 87% was very high and is in line with other reported results [6, 11, 20, 21]. It is assumable that stroke is a drastic event and is often accompanied by functional deficits that are well remembered in the course. However, previous studies used different ‘gold standards’ for comparison with the self-reported diagnoses making the direct comparison more difficult.

When using HID as a corresponding reference for self-reported data, the strongest agreement was seen for the diagnosis of AF with a sensitivity of 65% and a PPV of 78% in our study. These values were higher in comparison to the HUNT3-Study results, a Norwegian population-based cohort study that verified self-reported AF diagnoses by reviewing hospital and primary care medical records. The sensitivity of self-reported AF in the HUNT3 trial was 49.6% (PPV 66.2%) [7]. Conversely, Rix and colleagues validated the diagnoses of AF and atrial flutter recorded in the Danish National Patient Registry with hospital medical records. They found a higher PPV of 93.7% for the combined diagnosis of AF and/or atrial flutter [22]. However, the estimated prevalence of 5.6% (LIFE-Adult data) and 7.7% (HID), respectively, are comparatively high compared to other German population-based study results [23].

HF showed the lowest Cohen´s kappa value (0.26). This finding is consistent with other studies. Steinkirchner et al. also found a Cohen´s kappa of 0.26, similar to Hansen et al. with 0.24. Okura et al. reported a Cohen´s kappa at 0.46 [4, 9, 24]. The lowest sensitivity was seen for HF, with only 20% and a PPV of 56%. Prevalent HF cases were more than doubled when using HID as the data source (3.5% vs. 9.6%). In 2017, Camplain et al. assessed the accuracy of self-reported HF compared with physician-diagnosed HF in the ‘Atherosclerosis Risk in Communities (ARIC) Study’. Sensitivity of self-report was also low with 28–38%, while specificity at 96.4% (95% CI 96.1 to 96.8) was high, similar to our results [9]. It is assumable that the wording of “heart failure” is more unfamiliar than other known CVD. In addition, it is possible that the disease HF is little known and understood in the general population, as the symptoms are diverse, and the pathogenesis is complex. Moreover, HF is a syndrome rather than a diagnosis. Additionally, some HF-medication is equal to the treatment for hypertension or MI. Most affected people may do not even know about concomitant HF.

The sensitivity of self-reports for the diagnosis of MI ranges in other studies from 73% to 98% with various PPV [6, 11, 24]. Most of the previous studies used medical reports for validation of the self-reports. In contrast to our study, the sensitivity in the LIFE-study surveys was with 61% lower compared to others. However, our study used HID to verify self-reported diagnoses and direct comparison to other studies is, therefore, limited. In addition, the reported specificity and sensitivity may also produce biased results, even in the case of non-differential misclassification [25].

Briefly, the questions used in the LIFE-Adult baseline and follow-up surveys did not result in over-reporting. More importantly, participants are more likely to know about a stroke, AF, or MI diagnosis rather than a HF diagnosis. HF is a clinical syndrome that often combines several or complex symptoms compared to other conditions or can even be asymptomatic. Therefore, it may be essential to reconsider how to ask patients if they have a medical history of HF.

### The potential of misclassification of analyzed risk factors in self-reported data vs. HID

In population-based cohort studies, one of the main objectives is the assessment of risk factors predicting e.g., the development of disease, morbidity, or mortality. However, in epidemiological studies, bias can lead to inaccurate estimates of association, or over- or underestimation of risk parameters [26]. The multivariable logistic regression analyses in this study aimed to exemplarily observe the potential of misclassification of covariates based on the underlying data source. Our data did show that the association between potential risk factors and the observed disease differed based on the underlying data source. Steinkirchner et al. were able to determine a gender-specific difference in the agreement of the data in their comparison between self-report and general practitioner data. In their study, older males were associated with lower agreement [27]. Our analyses also revealed different results for female sex between self-reporting and HID. Future studies may need to take such potential influencing factors on disease understanding into account when gaining anamnestic data. This may allow a) a better specification of the extent of misclassification and b) data collection to be adapted more specifically to the individual subgroups.

However, it should also be critically noted that the wide confidence intervals of the odds ratios limit the interpretative power of the logistic regression analysis.

In this study, we acknowledge that while self-reports can be useful in specific contexts, they are often less reliable for accurately capturing disease events in most epidemiological studies. Although self-reporting may sometimes be the only feasible option, our results highlight its limitations compared to other sources of medical data, such as health insurance records. Future studies could refine methods to obtain more accurate estimates of disease burden from self-reports, for example, by developing weighted coefficients based on performance metrics from this and similar studies.

## Limitations

Due to the large number of health insurance companies in Germany and the difficulties in obtaining the necessary health data, it was only possible to evaluate data from two health insurance companies. Therefore, only a subgroup of the LIFE-adult cohort was represented at the time of the analysis. However, the study shows that linking the self-reporting with the HID is practicable and can be realised in compliance with data protection regulations. Furthermore German health insurance companies do not store all diagnoses for an arbitrary length of time [28]. Therefore, information about events that occurred a long time ago could be eventually lost.

The entry and coding of diagnoses are also partly based on the information provided by the individual, especially if the event occurred longer ago and was not diagnosed by the treating physician themself. Therefore, HID based data may not be completely free of errors and do not report with absolute certainty. It was not possible for us to check the diagnoses at a participant individual level, as we do not have access to the primary documents in the medical facilities. However, a validation with the information from the general practitioners is planned for further research. Nevertheless, we assumed that HID have a high degree of correctness, because the diagnoses are made by medical professionals. A ‘gold standard’ has not yet been found, as evidenced by the different comparators (e.g., general practitioner, administrative data) in the literature [3, 7, 27]. Although all participants were randomly selected from the resident register in Leipzig, there remains a participation selection bias. Initially, only 31% of the invited persons participated in the baseline investigation [29]. Among those, only 0.4% did not have any graduation. 80% had at least a general certificate of secondary education or a finished professional education, and 20% had a diploma. Therefore, no subgroup analyses for low vs. higher educational level were performed due the small sample size for subgroup analysis.

Furthermore, the results are not representative of other regions in Germany and cannot be generalised.

## Conclusion

The comparison of self-reported diagnoses with German health insurance-coded diagnoses showed differences in agreement across the four different cardiovascular diseases: stroke, myocardial infarction, atrial fibrillation, and heart failure. These results suggest that the verification of self-reported diagnoses in population-based assessments may be considered to reduce the potential for misclassification and errors when using self-reported data only.

## Supplementary Information

Supplementary Material 1.

## Data Availability

The datasets used and/or analyzed during the current study are available from the corresponding author on reasonable request.

## Abbreviations

AF: Atrial fibrillation
HF: Heart failure
MI: Myocardial infarction
HID: Health insurance data
CVD: Cardiovascular diseases
ICD- 10-GM: International Statistical Classification of Diseases and Related Health Problems 10th Revision-German modification
TP: True positive
FP: False positive
TN: True negative
FN: False negative
PPV: Positive predictive value
NPV: Negative predictive value
BMI: Body mass index
