# Supplementary material for: A comparison between the self-report of chronic cardiovascular diseases with health insurance data: insights from the population-based LIFE-Adult study
Source: Arch Public Health. 2025 May 7;83:124. doi: 10.1186/s13690-025-01606-3 (PMC12057059; doi:10.1186/s13690-025-01606-3)
Supplement: Supplementary file 1 — Supplementary Material 1. [file 13690_2025_1606_MOESM1_ESM.docx]

**A comparison between the self-report of chronic cardiovascular diseases with health insurance data: Insights from the population-based LIFE-Adult Study**

Samira Zeynalova^1,2† *^, Peter Worringen^1†^, Stefan Bassler^3^, Anja Martin^4^, Katrin Czech^4^, Lars Greulich^3^, Matthias Reusche^1,2^, Ute Enders^1^, Nigar Reyes^1^, Maryam Yahiaoui-Doktor^1^, Matthias Collier^5^,
Markus Loeffler^1,2†^, Tina Stegmann^6†^

**Supplementary material**

**Detailed Methods** Data linkage

**Supplemental Table 1.** ICD-10-GM coded diagnoses, which were used for the selection of the health insurance data.

**Supplemental Table 2.** Exemplarily analyses on misclassification errors in self-reported data based on the covariates age and sex. Results are given for sensitivity (in %) and the positive predictive value (PPV) with 95% confidence intervals, respectively.

**Detailed methods**

*Data linkage*

First, the names of the participants were replaced by an initial pseudonym in the study centre. Subsequently, the information was transmitted to an independent trust agency, which assigned a second pseudonym. The second pseudonym was then forwarded to the health insurance companies, who received the personal data of the subjects from the study centre. At the health insurance companies, the subjects' administrative data were linked to relevant information such as diagnoses, while all personal data such as name, date of birth, address, etc. were removed. The medical data were sent back to the data analysis department of the LIFE-Adult Study through the trust agency. Using this pathway, the evaluation team received the health insurance and self-disclosures for analysis via the trust agency without having access to the personal data.

**Supplemental Table 1**

| **Disease** | **ICD-10-GM Codes^*^** |
| --- | --- |
| **Stroke** | I60 Subarachnoid hemorrhage  I61 Intracerebral hemorrhage  I62 Other nontraumatic intracranial hemorrhage  I63 Cerebral infarction  I64 Stroke, not specified as hemorrhage or infarction  I69 Sequelae of cerebrovascular disease |
| **Atrial fibrillation** | I48 Atrial fibrillation and flutter  I48.0 Paroxysmal atrial fibrillation  I48.1 Persistent atrial fibrillation  I48.2 Chronic atrial fibrillation |
| **Heart failure** | I50 Congestive heart failure |
| **Myocardial infarction** | I21 Acute myocardial infarction  I22 Subsequent myocardial infarction |
| *All subcategories of each ICD-10 code were also included, respectively. | |

**Supplemental Table 2**

| **Disease** | **Subgroup (gender)** | **Sensitivity (%)** | **95% Confidence Interval** |
| --- | --- | --- | --- |
| **Myocardial infarction** | female | 21 | (5;37) |
|  | male | 59 | (45;72) |
| **Heart failure** | female | 20 | (11;30) |
|  | male | 23 | (13;32) |
| **Atrial fibrillation** | female | 69 | (53;84) |
|  | male | 71 | (58;85) |
| **Stroke** | female | 46 | (31;62) |
|  | male | 43 | (30;55) |
| **Disease** | **Subgroup (age, years)** | **Sensitivity (%)** | **95% Confidence Interval** |
| **Myocardial infarction** | <60 | 54 | (27;81) |
|  | ≥60 | 45 | (33;58) |
| **Heart failure** | <60 | 25 | (9;41) |
|  | ≥60 | 21 | (13;28) |
| **Atrial fibrillation** | <60 | 78 | (51;100) |
|  | ≥60 | 69 | (58;80) |
| **Stroke** | <60 | 55 | (33;77) |
|  | ≥60 | 41 | (31;52) |
| **Disease** | **Subgroup (gender)** | **PPP (%)** | **95% Confidence Interval** |
| **Myocardial infarction** | female | 63 | (29;96) |
|  | male | 79 | (66;91) |
| **Heart failure** | female | 48 | (30;66) |
|  | male | 53 | (35;71) |
| **Atrial fibrillation** | female | 59 | (43;74) |
|  | male | 53 | (40;66) |
| **Stroke** | female | 79 | (63;95) |
|  | male | 93 | (83;100) |
| **Disease** | **Subgroup (age, years)** | **PPP (%)** | **95% Confidence Interval** |
| **Myocardial infarction** | <60 | 88 | (65;100) |
|  | ≥60 | 74 | (60;88) |
| **Heart failure** | <60 | 64 | (35;92) |
|  | ≥60 | 48 | (34;62) |
| **Atrial fibrillation** | <60 | 64 | (35;92) |
|  | ≥60 | 54 | (44;64) |
| **Stroke** | <60 | 85 | (65;100) |
|  | ≥60 | 87 | (77;98) |
